# Dual-Channel Colloidal Gold-Based Immunochromatographic Test Strip for Rapidly Differentiating Between Two Major Groups of *Paracidovorax citrulli*

**DOI:** 10.3390/bios15030133

**Published:** 2025-02-22

**Authors:** Ling Sun, Yuanfei Xing, Xin Yang, Yanli Tian, Wenyao Zhang, Chen Zhang, Rui Fan, Weirong Gong, Jie Hu, Xiaolong Shao, Guoliang Qian, Baishi Hu, Limin Wang

**Affiliations:** 1 State Key Laboratory of Agricultural and Forestry Biosecurity, College of Plant Protection, Nanjing Agricultural University, Nanjing 210095, China; 2022102050@stu.njau.edu.cn (L.S.); 2023102033@stu.njau.edu.cn (X.Y.); tianyanli@njau.edu.cn (Y.T.); zhangwenyao1231@163.com (W.Z.); 19855132090@163.com (C.Z.); fanandrui@163.com (R.F.); 2021067@njau.edu.cn (X.S.); glqian@njau.edu.cn (G.Q.); hbs@njau.edu.cn (B.H.); 2 Chuzhou Academy of Agricultural Sciences, Chuzhou 239000, China; xyf0803@163.com; 3 Plant Protection and Quarantine Station of Jiangsu Province, Nanjing 210036, China; jsnjgwr@163.com (W.G.); hujie0925@126.com (J.H.)

**Keywords:** bacterial fruit blotch, genetic diversity, plastic cartridge, immunochromatographic test strip

## Abstract

Bacterial fruit blotch (BFB) caused by *Paracidovorax citrulli* is a severe threat to melon, watermelon, and other cucurbit crop production worldwide. The long-term adaptation of the pathogen to environmental conditions has resulted in substantial genetic diversity. In this study, we used *P. citrulli* strains from two groups as immunogens to obtain antibodies that were used to generate A.C1 and A.C2 colloidal gold immunochromatographic single test strips, which specifically identified group I and group II *P. citrulli* strains, respectively. We combined the A.C1 and A.C2 single test strips in a dual-channel plastic cartridge to construct a dual-channel colloidal gold immunochromatographic test strip able to distinguish between *P. citrulli* strains from two distinct groups. Test strip sensitivity reached 10^6^ CFU/mL under ideal conditions. Moreover, it was relatively stable, with no cross-reactions with strains of closely related genera. The dual-channel test strip developed in this study may provide farmers with a useful tool for managing BFB through the prompt implementation of quarantine procedures in the field.

## 1. Introduction

The Gram-negative bacterium *Paracidovorax citrulli* (*P. citrulli*) causes bacterial fruit blotch (BFB) [1,2], a devastating disease affecting cucurbit crops worldwide [3,4]. Because of its extremely destructive potential, *P. citrulli* is a serious threat to the global melon and watermelon industry, with BFB emerging as a significant commercial seed-borne disease of melon and watermelon [5]. After a serious epidemic in American watermelon fields in the late 1980s, BFB spread to many other regions [6,7]. *P. citrulli* can adhere to the seed surface or infiltrate the seed, leading to disease outbreaks due to seed-borne transmission [8,9]. The pathogen can spread quickly across nurseries and in the fields under favorable environmental conditions, such as high humidity and high temperatures, resulting in seedling blight or, later, fruit rot [10]. Typical symptoms of a *P. citrulli* infection of seedlings include water-soaked lesions on the cotyledons that later become necrotic lesions on the hypocotyls and cause seedling collapse and death [11]. Despite the implementation of some strict prevention and control measures, sporadic outbreaks of BFB in greenhouses and during fruit production continue to occur [12]. Because of its detrimental effects on crops, *P. citrulli* has been designated as a quarantine pest and is an agricultural phytosanitary pest in China [13] and other countries (European Plant Protection Organization global database; https://gd.eppo.int/datasheets/ (accessed on 20 November 2024) [14]. Thus, seedlings, agricultural products, and other sown materials are subject to strict quarantine regulations. For example, materials to be quarantined are strictly prohibited from entering protected areas. Moreover, quarantine treatments are required as soon as fruit blotch is detected in protected melon-producing areas to ensure the safety of non-infected areas.

There are differences among *P. citrulli* isolates according to pathogenicity tests, whole-cell fatty acid analyses, carbon source consumption, and DNA fingerprinting profiles [15,16]. In a previous study, on the basis of DNA fingerprinting and whole-cell fatty acid analyses, *P. citrulli* strains were divided into two major groups: group I comprises strains recovered mostly from non-watermelon hosts, whereas group II includes strains isolated primarily from watermelon seedlings and fruits [15]. In another study, pulse field gel electrophoresis and multilocus sequence typing results indicated *P. citrulli* isolates from melon and pumpkin belong to group I, while isolates from watermelon belong to group II [17]. Because group II strains are more sensitive to copper than group I strains, they may be controlled using copper-containing chemosynthetic drugs [18]. Hence, foliar-applied copper-based chemicals are commonly used in melon- and watermelon-producing areas; however, the excessive use of these chemicals has resulted in copper-tolerant isolates and environmental degradation [19]. Thus, an environmentally friendly, effective, and efficient method for preventing and controlling BFB infections in fields is required. Infections by group I and group II strains during the production of melon and other fruits have caused devastating economic losses. Accordingly, we focused our research on developing methods for easily differentiating and identifying strains in these two major groups. When BFB pathogen subtypes are quickly identified, the appropriate treatment may be applied, thereby decreasing pesticide use and increasing control efficiency.

There are several options for detecting *P. citrulli*, including semi-selective medium-based isolation [20,21], enzyme-linked immunosorbent assays (ELISAs) [22,23,24], immunoassays [25,26], and multiplex PCR [27] (Table 1). Some of these methods, such as semi-selective medium-based isolation and multiplex PCR, typically necessitate the collection of samples and delivery to a specific laboratory, which is both time-consuming and costly. When plants in the field are subjected to large-scale pathogen infections, disease detection becomes a labor-intensive and expensive process. Furthermore, in the event of a BFB outbreak, quarantine measures must be applied, especially during the first screening procedure, because BFB is a quarantine disease of domestic concern. Accordingly, there is an urgent need for a simple and cost-effective assay to quickly screen for *P. citrulli* under field conditions.

Immunochromatographic assays have the same underlying principle as ELISAs. The sole difference is that the immunological reaction for immunochromatographic assays takes place on chromatographic paper via capillary migration. Immunochromatography involves immunoreactions, with a labeled antibody serving as a visual marker to detect antibody-specific antigens in test suspensions. Most immunochromatographic techniques rely on antigen-specific antibodies [28]. Immunochromatographic assays are being developed and used for monitoring agricultural production, environmental conditions, and food safety. In terms of food safety, Wu et al. developed an immunochromatographic test strip (ITS) for detecting *Salmonella* strains; the sensitivity of the test strip for *Salmonella typhimurium* was 4 × 10^5^ CFU/mL, but it can also be used to detect 18 *Salmonella* strains, with no cross-reactions with 14 other food-borne pathogens [29]. Zhao et al. used cucumber green mottle mosaic virus capsid proteins produced in a prokaryotic system to generate monoclonal antibodies for an ITS with detection limits of up to 1:5000 (*w*/*v*) and no observed cross-reactions with tobacco mosaic virus or cucumber mosaic virus [30]. Zeng et al. used fluorescein isothiocyanate as a label to create a simple and fast immunoassay strip for detecting *P. citrulli*, with observable results obtained within 10 min and a detection limit of 10^6^ CFU/mL [25]. However, there are no published reports describing the use of an ITS applicable for the identification and taxonomic classification of *P. citrulli*. Hence, dual-channel test strips may be useful for the early detection of quarantine diseases, including BFB.

In this study, we hypothesized that quickly identifying and distinguishing between *P. citrulli* strains from groups I and II will make it feasible to screen for plant diseases under field conditions. We used isolated and purified *P. citrulli* strains to screen for highly specific antibodies, which were paired to develop an A.C1 single test strip for detecting group I strains and an A.C2 single test strip for detecting group II strains. Considering the possibility of mixed infections in a field, we used a dual-channel detection cartridge assembled in a dual-channel colloidal gold ITS to simultaneously detect and differentiate strains. The bioassay sensitivity of the test strip was 10^6^ CFU/mL, and the results were visible to the naked eye after 10 min. Moreover, after samples were added to dual-channel test wells, the test results precisely and rapidly revealed the subtype of the pathogen infecting the host. Thus, the test strip may be useful for promptly identifying pathogen types in a field, with no cross-reactions with its close relatives in our laboratory. Thus, test results may be used to determine the most appropriate treatment for a particular field. This ITS can efficiently and sensitively detect *P. citrulli*. Furthermore, it may be applicable for large-scale rapid analyses of samples under field conditions, with low technical requirements for testing personnel. ITS-based rapid field detection approaches may facilitate the prevention and control of infections by *P. citrulli* and its various subspecies.

## 2. Materials and Methods

### 2.1. Experimental Materials

The 15 group I and 20 group II *P. citrulli* strains, six strains from related genera, and SP2/0 cells used in this study are stored in the Plant Quarantine and Bacteriology Laboratory of Nanjing Agricultural University (Nanjing, China). Female BALB/c mice were obtained from the Center of Comparative Medicine of Yangzhou University (Yangzhou, China). Gold nanoparticles (40 nm) were prepared in our laboratory. After heating 100 mL of deionized water to its boiling point and adding 1 mL 1% HAuCl_4_, 1.2 mL 1% sodium citrate was added while stirring. After boiling for 5 min, the mixture was cooled and kept at 4 °C.

Sigma Chemical Co. (St. Louis, MO, USA) provided Freund’s complete/incomplete adjuvants, bovine serum albumin (BSA), and polyethylene glycol 1500. Gibco (Grand Island, NY, USA) provided Dulbecco’s Modified Eagle Medium, hypoxanthine thymidine, and hypoxanthine aminopterin thymidine. Tween-20 was supplied by Solarbio (Beijing, China), whereas an absorbent pad (H2), nitrocellulose (NC) membrane (PALL 120), binding pad (8965), sample pad (GF-08), and PVC base plate (MT101B) were acquired from Millipore (Billerica, MA, USA). Goat anti-mouse IgG conjugated with horseradish peroxidase was acquired from Boster. The ITS was prepared using the CM4000 Guillotine Cutter (BioDot, Irvine, CA, USA) and an XYZ-3060 dispensing platform. Thermo Electron (Waltham, MA, USA) provided a Forma Series II CO_2_ incubator. Relevant samples were stored at low temperatures (from −10 to −86 °C) in ultra-low temperature freezers (Meling, Hefei, China). General Electric (Fairfield, CT, USA) provided HiTrap™ Protein A HP and HiTrap™ Protein G HP.

### 2.2. Antibody Screening

#### 2.2.1. Strain Identification and Selection of Strains for Immunizations

*P. citrulli* strains and strains from related genera preserved in 20% glycerol in the laboratory were used for the inoculation of solid Luria-Bertani (LB) medium in plates. Each culture was activated by streaking. Of the 41 preserved strains, 35 were identified as *P. citrulli* and classified as group I or group II strains according to PCR amplification results, while the remaining six strains were from related genera (Table S1 in Supplementary Materials).

Nanjing Kengke Biological Co. (Nanjing, China) synthesized generic primers for *P. citrulli* [9] as well as specific primers for identifying group I and group II strains. For each strain, a single colony from a 48 h culture was suspended in 50 μL of sterilized distilled water, heated at 100 °C for 10 min, and used as the template for PCR amplification [31]. Specifically, PCR amplifications were carried out in 25 μL solutions comprising 2.5 μL 10× PCR Buffer (Mg^2+^-free), 2 μL Mg^2+^, 0.5 μL dNTP, 0.3 μL Taq polymerase, 0.3 μL forward and reverse primers, 1 μL template, and 18.4 μL sterile triple-distilled water. Standard *P. citrulli* strains were used as the positive control. The PCR program was as follows: pre-denaturation at 95 °C for 5 min; 30 cycles of denaturation at 95 °C for 30 s, annealing at 55 °C for 40 s, and extension at 72 °C for 30 s; 10 min at 72 °C and then 4 °C. Amplified products were analyzed by 1% agarose gel electrophoresis (1× TAE buffer; 15 min at 220 V). The gel was then photographed and analyzed using a digital image analyzer.

The strains BTL28 (group I) and XJL12 (group II) were selected for immunization on the basis of their growth rates in the LB medium. After comparing strain viability (i.e., growth), immunogens and encapsulants were prepared.

#### 2.2.2. Generation of Antibodies

*P. citrulli* BTL28 (group I) and XJL12 (group II) immunogens were mixed with an equal volume of Freund’s complete adjuvant, emulsified thoroughly, and injected (200 μL) intraperitoneally into each 7-week-old BALB/c female mouse. Three weeks after the first immunization, immunogens were mixed with an equal volume of Freund’s incomplete adjuvant, emulsified thoroughly, and injected (200 μL) intraperitoneally into each mouse. Subsequently, mice were immunized every 2 weeks (four injections in total). Beginning with the third immunization, venous blood was collected from the tail of each mouse at 7–10 days post-immunization to obtain antiserum. Venous blood collected from the tail of an unimmunized mouse was used as a negative control. The immune response was measured via an indirect ELISA. Following an 8000-fold dilution of the supernatant prepared from the collected blood, an OD_450_ of at least 1.0 was set as the threshold for a superior immune response (i.e., ELISA results: absorbance at OD_450_ ≥ 1.0; negative control absorbance at OD_450_ < 0.1). Mice with the highest potency and a substantial gradient ratio were selected for cell fusions and the production of monoclonal antibodies (i.e., hybridoma technique). Three days before cell fusions, the immunized mice were injected with the immunogen (without adjuvant) at the same dose as the initial immunization. The immunogens used to prepare monoclonal antibodies were also injected into New Zealand rabbits to produce polyclonal antibody sera. After being purified on a protein A antibody affinity column, the resulting antisera were aliquoted and stored at −80 °C.

A total of 10 positive cell lines specific for group I strains and 15 positive cell lines specific for group II strains were screened via animal immunization experiments and a hybridoma technique.

### 2.3. Preparation of Colloidal Gold-Labeled Monoclonal Antibodies

Gold nanoparticles with an average diameter of 40 nm were generated after reducing chloroauric acid using trisodium citrate [32]. Briefly, 0.2 M K_2_CO_3_ was used to adjust the pH of the colloidal gold solution to 9.0. Next, 20 μg antibody was added to 1.5 mL solution, which was then shaken and left undisturbed for 20 min. Filter-sterilized 10% BSA was then added (final concentration of 1%), after which the solution was thoroughly mixed for 5 min and incubated at room temperature for 1.5 h; the mixture was then centrifuged (10,000 rpm for 21 min at 4 °C). The supernatant was carefully removed and then the remaining sediment was redissolved in 0.01 M boric acid buffer supplemented with BSA (0.5%) and sucrose (6.0%). The conjugate was stored at 4 °C until used or spread on the binding pad.

### 2.4. Assembly of Colloidal Gold Immunochromatographic Single Test Strips

The ITS used in this study primarily consisted of a PVC base plate, sample pad (glass fiber), NC membrane, binding pad (glass fiber), and absorbent pad. Before being used, the binding pad was sliced into 5 mm wide strips, moistened with a sealing solution, and dried at 37 °C in a vacuum oven. It was then sprayed with a gold-labeled antibody solution (predetermined dilution) and dried for 4 h at room temperature. An XYZ-3060 dispensing platform was used to immobilize the capture and control antibodies on the NC membrane test line (T-line) and control line (C-line), respectively. Finally, the prepared sample pads, binding pads, NC membranes, and absorbent pads were attached to the PVC substrate (60-mm gauge) in the proper order, with a 2 mm overlap between each component.

### 2.5. Optimization of Working Conditions for Colloidal Gold Immunochromatographic Single Test Strips

In addition to storage and transport conditions, other factors influencing the accuracy and stability of the dual-channel colloidal gold ITS include the operating settings. Optimizing the test strip parameters is crucial for increasing test strip validity and decreasing inaccuracies across batches and within batches. The optimization of the test strip working conditions mainly focused on the antibody quantity, pH of the chromatographic solution, salt ion concentration, and surfactant concentration in the chromatographic solution.

### 2.6. Preparation of Samples

Test strips were analyzed using genuine samples and replicated field conditions to further assess test strip utility and produce useful results. Healthy melon seeds were sown and the resulting seedlings were cultivated in a greenhouse. After the seedlings had 7–8 true leaves, they were used for testing. First, to evaluate the effects of leaves on the test strips, healthy leaves (0.05, 0.1, 0.15, and 0.2 g) were placed in a grinding bag, after which 2 mL carbonate buffer solution (*P. citrulli* concentration of 1 × 10^8^ CFU/mL) was added to each grinding bag. The buffer alone served as a negative control. The abrasive side of a single-sided abrasive bag was folded in the center and then the sample was placed on the folded surface for a light abrasion. After grinding, 100 μL of solution was added dropwise into the spiked end of the test strip, which was laid flat. The strip was checked for detectable bands within 10 min. Second, to simulate leaf infections under natural conditions, healthy leaves were injected with *P. citrulli* group I or group II strains or related genera (1 × 10^8^ CFU/mL) and examined 1 week later for disease symptoms. Healthy leaves injected with buffer alone were used as a negative control. Infected leaves were placed in a grinding bag, after which 2 mL buffer alone was added. Leaves were ground and added to test strips as described above. The test strips were left undisturbed at room temperature for 10 min before being examined for detectable bands. This analysis was performed three times.

## 3. Results

### 3.1. Establishment of A.C1 and A.C2 Immunochromatographic Single Test Strips

In this study, ITSs were developed to accurately determine whether target pathogens were present in a test sample. Using two antibodies specific for group I and group II strains, we produced A.C1 single test strips that identify BTL28 (group I), and A.C2 single test strips that identify XJL12 (group II). For the A.C1 single test strip, a T-line was obtained for the BTL28 bacterial solution but not for the XJL12 bacterial solution or the detection buffer (Figure S1A). The results of the paired antibody test in the A.C2 single test strip showed that a T-line was obtained for the XJL12 bacterial solution but not for the detection buffer or the BTL28 bacterial solution (Figure S1B). The concentrations of the BTL28 and XJL12 bacterial solutions were 1 × 10^8^ CFU/mL.

#### 3.1.1. Optimization of the A.C1 and A.C2 Immunochromatographic Single Test Strips

Many factors influence ITS performance, including the pH of the colloidal gold solution [33], which can be too high or too low, leading to antibody denaturation. Antibody concentration is also an important factor [34], with excessive amounts wasting antibodies and resulting in false positives. Tween-20, a nonionic surfactant, inhibits nonspecific antibody binding while leaving proteins intact. Additionally, moderate quantities of Tween-20 can improve the capture of gold-labeled antibodies on NC membranes [30]. Therefore, we considered these three factors while optimizing single-channel test strip working conditions.

##### Optimization of Chromatographic Solution pH

When analyzing samples using test strips, the pH of the chromatographic solution influences the structure and specific binding reaction of the antigen and antibody. Thus, an inappropriately acidic or alkaline test condition can adversely affect the results. In this study, test conditions were optimized at pH 3.0, 5.0, 7.0, 9.0, and 11.0. The findings revealed that a low pH value (acidic) will lead to a negative test strip detection result, whereas a high pH value (excessively alkaline) may disrupt the antibody structure, with detrimental effects on detection. The optimal pH for the chromatographic solutions in the A.C1 and A.C2 single test strips was 9.0 (i.e., clear positive result and no negative result) (Table S2).

##### Optimization of the T-Line

To determine the optimal capture antibody concentration for the T-line, five concentrations (1.0, 1.5, 2.0, 2.5, and 3.0 mg/mL) were prepared. The difference in coloration between the positive and negative results was most visible when the concentration was 2.5 mg/mL. More specifically, a highly intense coloration was detected for the positive result, which was in contrast to the undetectable coloration for the negative result (Table S2).

##### Optimization of Surfactants

On the basis of hydrophilicity, Tween-20 (non-ionic surfactant) was selected for optimization. Five concentrations were set (0.1%, 0.3%, 0.5%, 1%, and 2%). A 0.5% Tween-20 concentration produced the darkest positive results, with undetectable negative results (Table S2).

#### 3.1.2. Analytical Evaluation of the A.C1 and A.C2 Immunochromatographic Single Test Strips

##### Sensitivity of the A.C1 and A.C2 Single Test Strips

**Figure 1:**
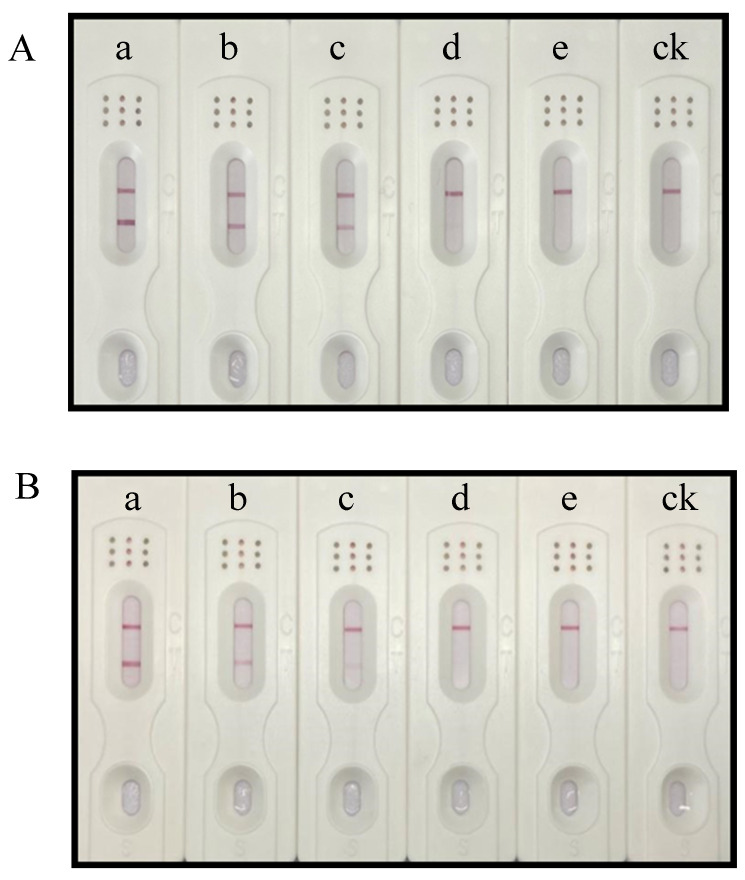
Sensitivity of A.C1 and A.C2 test strips for single detection. (**A**) Sensitivity analyses of the A.C1 single test strip for detecting BTL28 (group I). (**B**) Sensitivity analyses of the A.C2 single test strip for detecting XJL12 (group II). a–e, 1 × 10^8^ CFU/mL, 1 × 10^7^ CFU/mL, 1 × 10^6^ CFU/mL, 1 × 10^5^ CFU/mL, and 1 × 10^4^ CFU/mL, respectively; ck, carbonate buffer solution (CBS, 0.05 M, pH 9.0).

To determine the visual detection limits of the A.C1 and A.C2 single test strips, positive test solutions for the strains BTL28 (group I) and XJL12 (group II) were prepared at different bacterial concentrations (1 × 10^8^, 1 × 10^7^, 1 × 10^6^, 1 × 10^5^, and 1 × 10^4^ CFU/mL). Single test strips under optimal conditions were used. Color intensity was measured 10 min after samples were added. For the A.C1 single test strip, a bright red band was detected for the positive result, with no detectable band for the negative result, at BTL28 (group I) concentrations as low as 1 × 10^5^ CFU/mL (Figure 1A). For the A.C2 single test strip, a light red band was detected for the positive result, with no detectable band for the negative result, at an XJL12 (group II) concentration of 1 × 10^6^ CFU/mL (Figure 1B).

##### Stability of the A.C1 and A.C2 Single Test Strips

Single test strips were collected for analyses on a regular basis within a period comprising a 120-day period. Each batch was kept separately at room temperature in foil bags with a desiccant under dry conditions. Each pair of test strips was checked using 0.05 M CBS, BTL28 (group I), and XJL12 (group II). The A.C1 and A.C2 single test strips produced highly stable and valid results for at least 4 months (Figure S2).

##### Specificity of the A.C1 and A.C2 Single Test Strips

The A.C1 and A.C2 single test strips were used to detect 41 strains. The A.C1 single test strip produced positive and negative results for the 15 group I strains and 20 group II strains, respectively. Conversely, the A.C2 single test strip generated positive and negative results for the 20 group II strains and 15 group I strains, respectively. Six strains from related genera were used to further assess the specificity of the A.C1 and A.C2 single test strips. Primers specific for group II strains [9] and universal primers for group I and group II strains [31] were used for PCR analysis of the experimental strains. The PCR results for the *P. citrulli* strains were consistent with the test strip results (Table S1). Moreover, there were no detectable cross-reactions. Diagnostic tests are crucial clinical tools [35]. The specificity of the test strips was further verified by the consistency between the PCR results and the test strip results.

### 3.2. Development of a Dual-Channel Immunochromatographic Test Strip

Considering the possibility that susceptible plants in natural environments may be infected by a mixture of group I and group II strains, we created a dual-channel ITS that can detect strains from both groups. A more precise identification of pathogens may facilitate a more focused treatment for preventing and managing infections.

#### 3.2.1. Characterization of a Dual-Channel Immunochromatographic Test Strip

**Figure 2:**
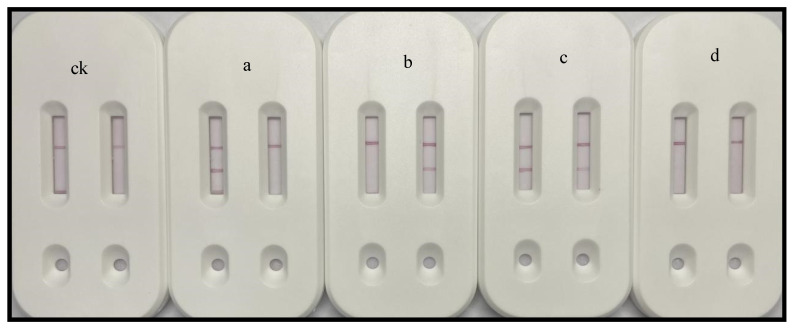
Characterization of dual-channel test strips (A.C1 single test strip in the left channel and A.C2 single test strip in the right channel). a: Detection of a group I strain. b: Detection of a group II strain. c: Detection of a mixture comprising group I and group II strains. d: Detection of the related genera. ck: Detection of the CBS.

The dual-channel ITS, which consisted of an A.C1 single test strip in the left channel and an A.C2 single test strip in the right channel, was used to analyze group I and group II *P. citrulli* strains, strains from related genera, and strains from both groups. For the analysis of group I strains, positive and negative results were obtained for the left and right channels, respectively. In contrast, for the analysis of group II strains, negative and positive results were obtained for the left and right channels, respectively. When a mixed bacterial solution comprising group I and group II strains was used, both the left and right channels produced positive results. However, both channels produced negative results when the strains from related genera were analyzed (Figure 2).

#### 3.2.2. Detection of Randomly Mixed Bacterial Solutions

**Figure 3:**
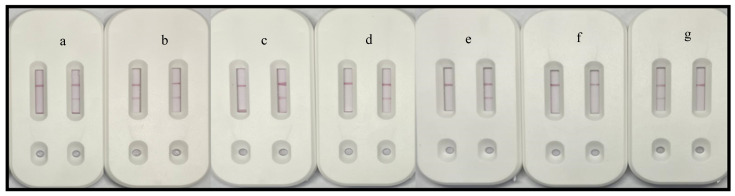
Detection of mixed strains using dual-channel test strips. a, 1 + 2; b, 1 + 5; c, 6 + 7; d, 4 + 7; e, 3 + 7; f, 2+ 7; g, 2 + 8.

**Figure 4:**
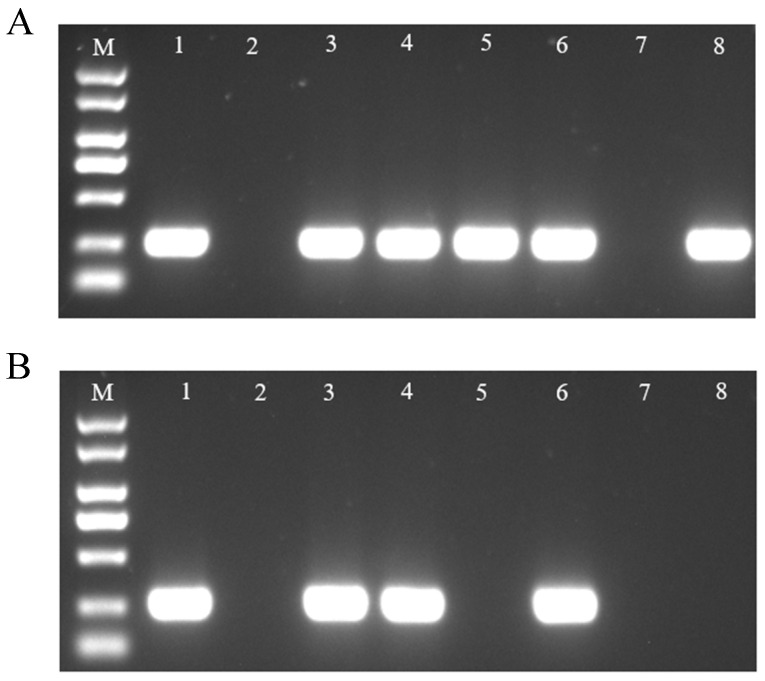
PCR amplification to verify eight strains. (**A**) Verification of eight randomly selected strains via PCR amplification using universal primers for group I and group II strains. Amplification results revealed that strains 2 and 7 are related genera (i.e., no bands), whereas strains 1, 3, 4, 5, 6, and 8 are group I or group II strains. (**B**) Verification of eight randomly selected strains via a PCR amplification using primers specific for group II strains. In addition to the lack of bands for strains 2 and 7, there were also no bands for strains 5 and 8. This suggests that strains 5 and 8 are group I strains, whereas strains 1, 3, 4, and 6 are group II strains.

Because of the substantial diversity in the flora under natural conditions and the possibility of mixed infections, we conducted experiments under simulated field conditions. We randomly selected eight strains from among the 41 strains (strains 1–8), which were then combined in random pairs to evaluate the performance of the dual-channel test strip and its utility for detecting strain types. The combined bacterial solutions were analyzed individually using the dual-channel test strip. According to the results (Figure 3), strains 2 and 7 are from related genera, strains 5 and 8 are from group I, and strains 1, 3, 4, and 6 are from group II. The eight randomly selected strains were validated by PCR. The results of PCR amplifications were in accordance with the dual-channel test strip results (Figure 4A, B).

#### 3.2.3. Evaluation of Leaf Effects on Test Strips

Plant leaves contain pigments as well as a number of complex substances that tend to interfere with the performance of test strips [36]. Therefore, we analyzed the potential effects of leaves on the ability of the test strips to identify bacteria, with implications for the future utility of the test strips for screening fields for *P. citrulli* strains from the two major groups. Healthy leaves (0.05, 0.1, 0.15, and 0.2 g) were ground in 2 mL working buffer (0.05 M CBS, pH 9.0) before being tested. The test strips had a clear chromatographic background. In addition, the chromatographic analysis was completed relatively quickly, with no false positives when the leaf mass was 0.05 or 0.1 g. A leaf mass of approximately 0.1 g was selected for the actual sample analysis because it may influence the ability of the test strips to detect bacteria. A bacterial solution mixed with 0.1 g healthy leaves served as the positive control, whereas the buffer alone (i.e., no leaves) served as the negative control. The test results are provided in Table 2. Notably, tests involving healthy leaves produced negative results, suggesting the leaf mass had no obvious effect on the test strips. Thus, leaves are appropriate test materials for the dual-channel test strip.

**Figure 5:**
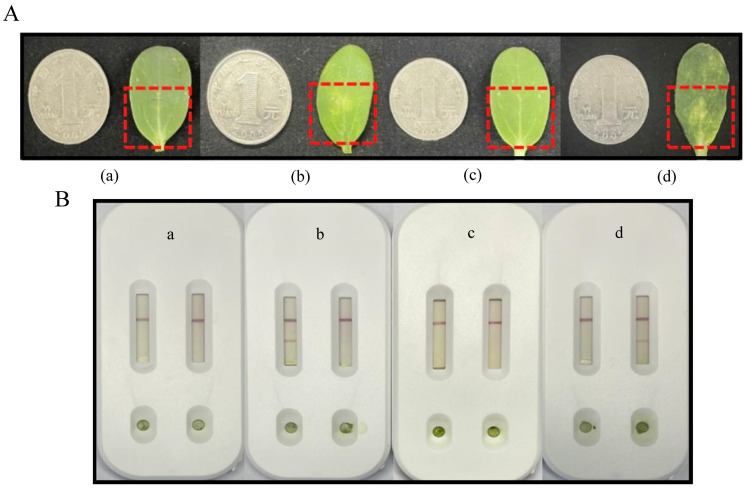
Testing of inoculated samples using dual-channel test strips. (**A**) Inoculated samples. (**B**) Dual-channel test strip results for inoculated samples. a, CBS; b, BTL28; c, ChaAaa; d, XJL12 injected separately.

Inoculated samples (Figure 5A) were analyzed using the dual-channel test strip, which precisely identified the corresponding bacteria (Figure 5B).

## 4. Conclusions

Using BTL28 (group I) and XJL12 (group II) for immunizations, we screened numerous cell lines secreting highly specific antibodies, which were used to produce A.C1 and A.C2 colloidal gold immunochromatographic single test strips. We integrated the two single test strips in a dual-channel colloidal gold ITS. The test strips accurately and rapidly identified and differentiated between two groups of pathogenic bacteria. Moreover, the test strips were highly specific and stable, with a sensitivity of up to 10^6^ CFU/mL. The antibodies used in the dual-channel test strip are highly stable and homogeneous in terms of physicochemical properties. Furthermore, the test strips developed in this study generated reproducible results, making them applicable for on-site disease screening and diagnosis.

## Figures and Tables

**Table 1:** Comparison of different methods for detecting *P. citrulli*.

Method	LOD ^a^ (CFU/mL)	Detection Time	Identifying Objects	Stability	Reference
IFS	1 × 10^6^	10 min	8 strains of *P. citrulli* (unclassified subtypes)	4 months	[25]
MIA	1 × 10^8^	2.5 h	8 strains of *P. citrulli* (unclassified subtypes)	-	[26]
DAPS-ELISA	5 × 10^5^–1 × 10^6^	2–5 h	16 strains of *P. citrulli* (unclassified subtypes)	-	[24]
Multiplex PCR assay	1 × 10^5^	5 h	Type 1: 82 strains of *P. citrulli* Type 2: 47 strains of *P. citrulli*	-	[27]
ITS	1 × 10^6^	10 min	Group I: 15 strains of *P. citrulli* Group II: 20 strains of *P. citrulli*	4 months	This study

^a^ Minimum detection limit.

**Table 2:** Leaf effects on dual-channel test strips.

Factor		Dual-Channel Test Strips			
		A.C1		A.C2	
		Test Results ^b^	Background of Chromatography ^c^	Test Results ^b^	Background of Chromatography ^c^
Healthy leaf (g)	0.05	−	/	−	/
	0.1	−	/	−	/
	0.15	−	*	−	*
	0.2	−	**	−	**
CBS	0.05 M, pH 9.0	−	/	−	/
Positive control ^a^	BTL28 + 0.1 g healthy leaf	+	/	−	/
	XJL12 + 0.1 g healthy leaf	−	/	+	/

^a^ Bacterial concentration was adjusted to 1 × 10^8^ CFU/mL in 0.05 M CBS. For testing, 2 mL bacterial solution was added to 0.1 g healthy leaves before grinding. ^b^ “−” and “+” represent negative and positive results, respectively. ^c^ “*” indicates a green chromatographic background, with “**” representing a more intense coloration than “*”; “/” indicates a clear background (i.e., no greenish coloration).

## Data Availability

The data presented in this study are available on request from the corresponding author. The data are not publicly available due to ethical constraints.

## Supplementary Materials

The following supporting information can be downloaded at: https://www.mdpi.com/article/10.3390/bios15030133/s1, Table S1: Bacterial strains used in this study and detection results by PCR and test strips; Table S2: Effect of different parameters on the A.C1 and A.C2 single test strip results; Figure S1: Characterization of the A.C1 and A.C2 test strips for single detection; Figure S2: Stability of the A.C1 and A.C2 test strips for single detection.
