# Supplementary material for: Dual-Channel Colloidal Gold-Based Immunochromatographic Test Strip for Rapidly Differentiating Between Two Major Groups of Paracidovorax citrulli
Source: Biosensors (Basel). 2025 Feb 22;15(3):133. doi: 10.3390/bios15030133 (PMC11940258; doi:10.3390/bios15030133)
Supplement: Supplementary file 1 [file biosensors-15-00133-s001.zip › biosensors-3437775-supplementary.pdf]

Dual-Channel Colloidal Gold-Based Immunochromatographic Test Strip for Rapidly Differentiating Between Two Major Groups of *Paracidovorax citrulli*

Ling Sun [1,†], Yuanfei Xing [2,†], Xin Yang [1], Yanli Tian [1], Wenyao Zhang [1], Chen Zhang [1], Rui Fan [1], Weirong Gong [3], Jie Hu [3], Xiaolong Shao [1], Guoliang Qian [1], Baishi Hu [1] and Limin Wang [1,*]

[1] State Key Laboratory of Agricultural and Forestry Biosecurity, College of Plant Protection , Nanjing Agricultural University, Nanjing 210095, China; 2022102050@stu.njau.edu.cn (L.S.); 2023102033@stu.njau.edu.cn (X.Y.); tianyanli@njau.edu.cn (Y.T.); zhangwenyao1231@163.com (W.Z.); 19855132090@163.com (C.Z.); fanandrui@163.com (R.F.); 2021067@njau.edu.cn (X.S.); glqian@njau.edu.cn (G.Q.); hbs@njau.edu.cn (B.H.)

[2] Chuzhou Academy of Agricultural Sciences, Chuzhou 239000, China; xyf0803@163.com

[3] Plant Protection and Quarantine Station of Jiangsu Province, Nanjing 210036, China; jsnjgwr@163.com (W.G.); hujie0925@126.com (J.H.)

* Correspondence: wlm@njau.edu.cn

† These authors contributed equally to this work.

Supporting

Table S1. Bacterial strains used in this study and detection results by PCR and test strips

No.	Bacterial strains	Group	PCR results		Test strips results	
			universal primers	specific primers	A.C1	A.C2
1	*Paracidovorax citrulli* XJL03	I	+	–	+	–
2	*P. citrulli* XJL16	I	+	–	+	–
3	*P. citrulli* XJL22	I	+	–	+	–
4	*P. citrulli* njl03	I	+	–	+	–
5	*P. citrulli* njl05	I	+	–	+	–
6	*P. citrulli* njl15	I	+	–	+	–
7	*P. citrulli* njl23	I	+	–	+	–
8	*P. citrulli* BTL09	I	+	–	+	–
9	*P. citrulli* BTL16	I	+	–	+	–
10	*P. citrulli* BTL26	I	+	–	+	–
11	*P. citrulli* BTL27	I	+	–	+	–
12	*P. citrulli* BTL28	I	+	–	+	–
13	*P. citrulli* BTL29	I	+	–	+	–
14	*P. citrulli* BTL75	I	+	–	+	–
15	*P. citrulli* 152	I	+	–	+	–
16	*P. citrulli* 1ST	II		+	–	+
17	*P. citrulli* 2ST	II		+	–	+
18	*P. citrulli* 3ST	II		+	–	+
19	*P. citrulli* 4ST	II		+	–	+
20	*P. citrulli* 5ST	II		+	–	+
21	*P. citrulli* 6ST	II		+	–	+
22	*P. citrulli* 7ST	II		+	–	+
22	*P. citrulli* 7ST	II		+	–	+
23	*P. citrulli* 8ST	II		+	–	+
24	*P. citrulli* 9ST	II		+	–	+
25	*P. citrulli* 10ST	II		+	–	+
26	*P. citrulli* 11ST	II		+	–	+
27	*P. citrulli* 12ST	II		+	–	+

28	*P. citrulli* 13ST	II		+	_	+
29	*P. citrulli* 14ST	II		+	_	+
30	*P. citrulli* 15ST	II		+	_	+
31	*P. citrulli* Aac-0-16	II		+	_	+
32	*P. citrulli* Au-9	II		+	_	+
33	*P. citrulli* njf01	II		+	_	+
34	*P. citrulli* njf02	II		+	_	+
35	*P. citrulli* XJL12	II		+	_	+
36	*Acidovorax avenae* ChaAaa	related genera	_		_	_
37	*A. avenae* FC358	related genera	_		_	_
38	*Pantoea herbicola* 0084	related genera	_		_	_
39	*Pseudomonas syringae* njl16	related genera	–		–	–
40	*Acidovorax konjaci* ATCC33996	related genera	–		–	–
41	*Acidovorax cattleyae* ATCC10200	related genera	–		–	–

“+”represents a positive result, “_”represents a negative result. Group II strains can be specifically detected by specific primers, universal primers can detect group I and group II strains.

Table S2. Effect of different parameters on the A.C1 and A.C2 single test strip results

**Optimization Factors**		**A.C1**		**A.C2**	
		Buffer Solution [a]	BTL28 [b]	Buffer Solution [a]	XJL12 [b]
pH	3.0	+	+	+	+
	5.0	+	++	+	+
	7.0	+	++	-	++
	9.0	-	+++	-	+++
	11.0	-	+	-	+
Test line	1.0 mg/mL	- [c]	+	-	+
	1.5 mg/mL	-	++	-	+
	2.0 mg/mL	-	++	-	++
	2.5 mg/mL	-	+++	-	+++
	3.0 mg/mL	+	+++	+	+++
Tween-20(%)	0.1	-	+	-	+
	0.3	-	++	-	+
	0.5	-	+++	-	++
	1	+	+++	-	++
	2	+	+++	+	+++

[a] Carbonate buffer solution (CBS).

[b] Detection of bacteria concentration is 1×10^8 CFU/mL.

[c] The presented data indicate the color development of the bands in the test strip which compared with the detection effect of the immunogenic strain. (“+” represents the depth of the A.C1 and A.C2 single test strip's detection line; “+++” represents the darkest color of the detection line; “++” is darker than “+”; “-” represents no color.)

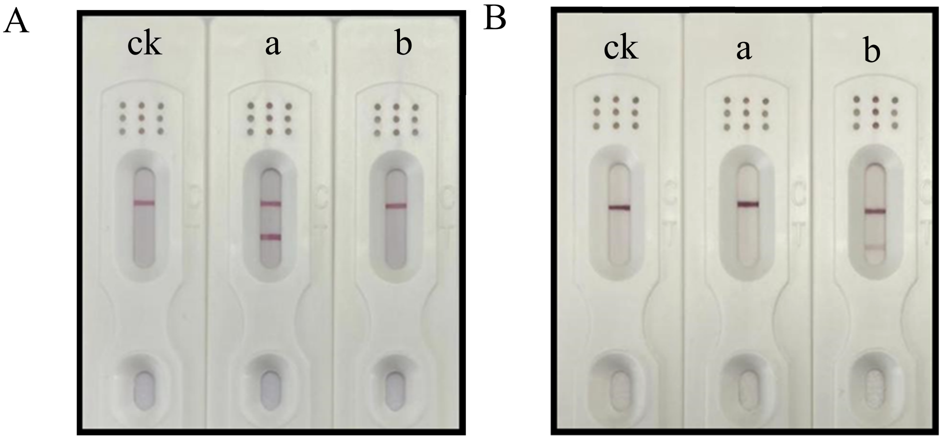

Figure S1. Characterization of the A.C1 and A.C2 test strips for single detection. A, The detection of BTL28 (group I) by using the A.C1 single test strip. B, The detection of XJL12 (group II) by using the A.C2 single test strip. a, BTL28; b, XJL12; ck, CBS (0.05 M, pH 9.0).

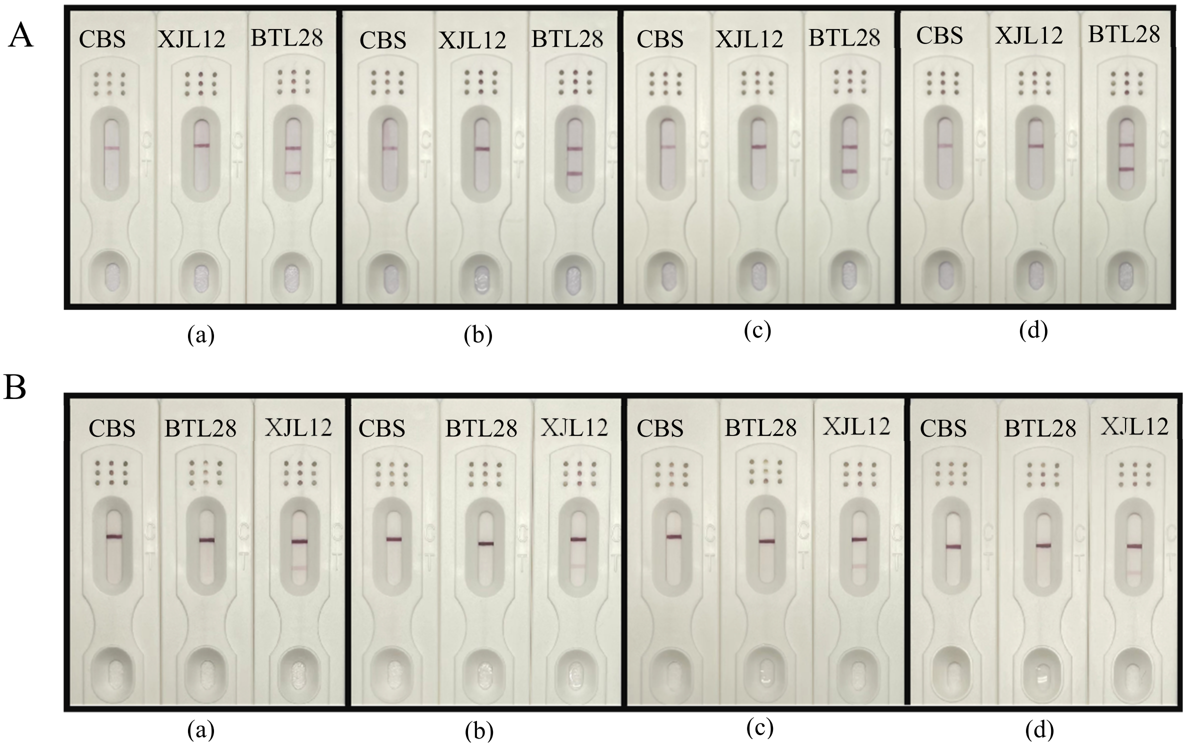

Figure S2. Stability of the A.C1 and A.C2 test strips for single detection. A, The stability assessment of the A.C1 single test strip. B, The stability assessment of the A.C2 single test strip. a, 15 days; b, 30 days; c, 60 days; d, 120days.
